# Two new species of the genus *Saigona* Matsumura (Hemiptera, Fulgoromorpha, Dictyopharidae) from China

**DOI:** 10.3897/zookeys.1054.67004

**Published:** 2021-08-04

**Authors:** Yan-Li Zheng, Thierry Bourgoin, Lin YangXiang-Sheng Chen1, Xu-Qiang Luo, Guang-Jie Luo

**Affiliations:** 1 Institute of Entomology, Guizhou University, The Provincial Key Laboratory for Agricultural Pest Management of Mountainous Region, Guiyang, Guizhou, 550025, China; 2 School of Geography and Resources, Guizhou Education University, Guiyang, Guizhou, 550018, China; 3 Guizhou Provincial Key Laboratory of Geographic State Monitoring, Guiyang, 550018, China; 4 Institute of Environmental Resources and Disaster, Guizhou Education University, Guiyang, Guizhou, 550018, China; 5 Institut Systématique, Evolution, Biodiversité (ISYEB), UMR 7205 MNHN-CNRS-Sorbonne Université-EPHE-Univ. Antilles, Museum National d’Histoire Naturelle, 75005, Paris, France

**Keywords:** Fulgoroidea, Oriental region, planthopper, taxonomy

## Abstract

Two new species of the genus *Saigona* Matsumura, 1910, *S.baiseensis* Zheng & Chen **sp. nov.** and *S.maculata* Zheng & Chen **sp. nov.**, from China (Guanxi) are described and illustrated. A revised identification key to the 16 species of *Saigona* is provided. 15 species of the genus are known from China only.

## Introduction

**Figure 1.**
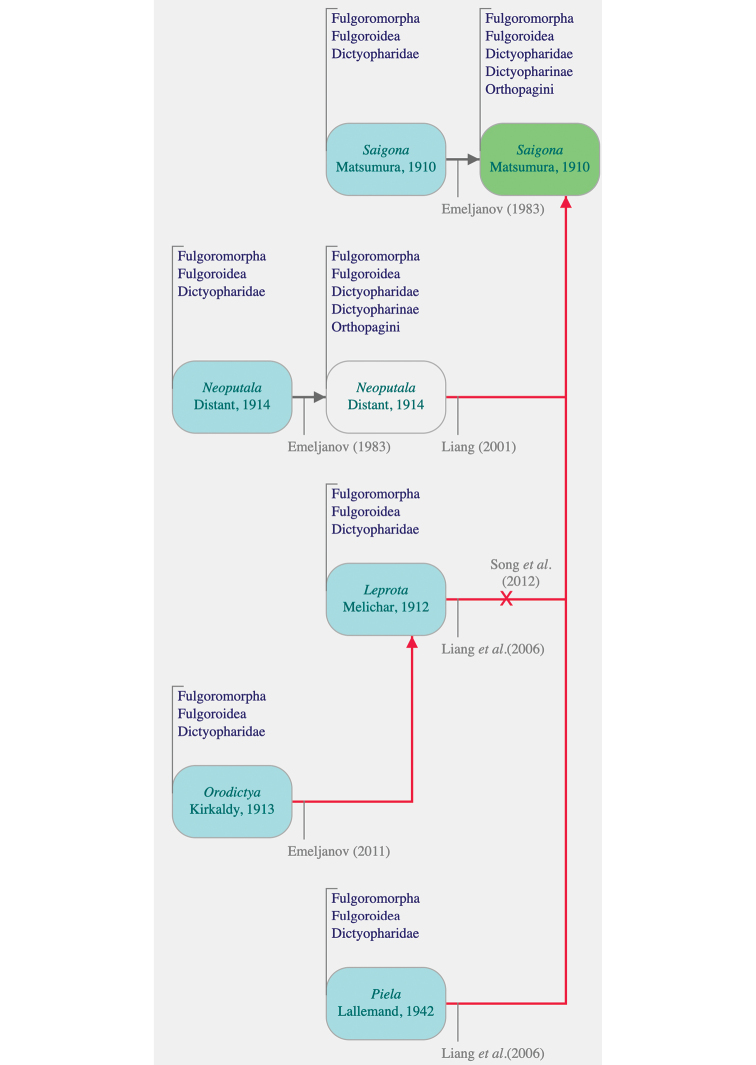
Taxonomic history of the genus *Saigona* Matsumura, 1010 (uploaded from Bourgoin 2021). For each synonymized genus, their protonyms are in blue boxes, and the current valid name of the genus is in green. Red vertical arrows illustrate synonymy and the red cross the *status revivisco* of the genus *Leprota* Melichar, 1912.

The planthoppers of the family Dictyopharidae Spinola, 1839 (Hemiptera, Fulgoromorpha) currently groups 738 species in 160 extant and extinct genera (Bourgoin 2021). They are currently divided into two subfamilies, Dictyopharinae Spinola, 1839 and Orgeriinae Fieber, 1872, and 19 tribes (Muir 1923; Metcalf 1946; Song et al. 2018; Bourgoin 2021). The genus *Saigona* Matsumura, 1910 was first established by Matsumura (1910) based on *Dictyophora* [sic] *ishidae* Matsumura, 1905 from Japan and later classified in Dictyopharinae (Orthopagini) by Emeljanov (1983). The genus has a rather complex taxonomic history, with several genera synonymized with it and 14 described species. The genus *Neoputala* Distant, 1914 was the first synonymized by Liang (2001), followed by *Leprota* Melichar, 1912 and *Piela* Lallemand, 1942 (Liang and Song 2006). Eight species had been recognized in the genus at that time. Subsequently, Zheng and Chen (2011) and Zheng et al. (2014) added one, then four, new species, all from China. In 2011, Emeljanov (2011) synonymized genus *Orodictya* Kirkaldy, 1913 with *Leprota*, the later genus being resurrected from the synonymy with *Saigona* and re-established as a valid genus by Song et al. (2012). A summary of all these changes is provided in Figure 1.

While sorting and identifying recently collected specimens, two new species, *S.baiseensis* sp. nov. and *S.maculata* sp. nov., were discovered from Guangxi province, China, and they are described here. *Saigona* now includes 16 species, with 15 of them endemic to China. A revised identification key to all species is provided, and the rather restricted distribution of this rather prolific genus is briefly discussed.

## Materials and methods

The morphological terminologies follow Yang and Yeh (1994) for the head and body, Bourgoin et al. (2015) for the wing venation, Bourgoin (1987, 1993), and Yang and Yeh (1994) for male and female genitalia, respectively. Biogeographical realms terminology follows Holt et al. (2013). The specimens examined have been deposited in the Institute of Entomology, Guizhou University, Guiyang, China (**GUGC**). Dry specimens were used for the descriptions and illustrations. Genital segments of the specimens were macerated in boiling solution of 10% NaOH, transferred to preparations of glycerin jelly, and examined under a Leica MZ12.5 stereomicroscope. Photographs of adult habitus were obtained using a Keyence VHX-1000 system. Illustrations were scanned with Canon Cano Scan LiDE 200 and imported into Adobe Photoshop CS6 for labeling and composition of figures.

The following abbreviations are used in the text:

**BL** body length (from apex of cephalic process to tip of forewings);

**HL** head length (from apex of cephalic process to base of eyes);

**HW** head width (including eyes);

**FWL** orewing length.

The usual standardized notation is used for the wing venation as follow:

**A1** first anal vein; bc, basal cell;

**MP** media posterior;

**CuA** cubitus anterior;

**CuP** cubitus posterior;

**RP** radius posterior;

**Pcu** postcubitus;

**ScP** subcosta posterior.

## Taxonomy

### Family Dictyopharidae Spinola, 1839

**Subfamily Dictyopharinae Spinola, 1839**

#### Tribe Orthopagini Emeljanov, 1983

##### Genus Saigona

Taxon classificationAnimaliaHemipteraDictyopharidae

Matsumura, 1910

85BA0CB7-18A9-5181-B9FC-7D31D2BCF911

###### Type species.

*Dictyophora* [sic] *ishidae* Matsumura, 1905 [= *Almanaussuriensis* Lethierry, 1878], by subsequent designation of Melichar (1912: 50).

*Neoputala* Distant, 1914: 412; Metcalf 1946: 78. Type species: *Neoputalalewisi* Distant, 1906 (by monotypy) [not *Neoputalacapitata* Distant, 1914, as stated by Liang (2001: 236)], synonymised by Liang (2001: 236).

*Leprota* Melichar, 1912: 91; Metcalf 1946: 74. Type species: *Dictyophora* [sic] *fulgoroides* Walker, 1858, by original designation and monotypy. synonymized by Liang and Song (2006: 28).

*Piela* Lallemand, 1942: 72. Type species: *Pielasingularis* Lallemand, 1942, by original designation and monotypy. synonymized by Liang and Song (2006): 28.

*Orodictya* Kirkaldy, 1913: 16. Type species: *Orodictyamonticola* Kirkaldy, 1913; by original designation. Synonymized by Emeljanov (2011: 1144).

*Leprota* Melichar, 1912: 91. Type species: *Leprotamelichari* Fennah, 1963; *status revivisco* according to Song et al. (2012: 218).

*Saigona* Matsumura, 1910: 110; Melichar 1912: 28, 50; Metcalf 1946: 47; Nast 1972: 84; Chou et al. 1985: 63; Anufriev and Emeljanov 1988: 482; Emeljanov 1993: 70; Liang 2001: 235; S. Matsumura 1941: 163; Liang and Song 2006: 28; Zheng and Chen 2011: 542; Zheng et al. 2014; Song et al. 2016: 350, 2018: 3.

###### Diagnosis.

This species can be distinguished from other dictyopharid planthoppers by the combination of the following diagnostic characters: (1) general color ochraceous or fuscous; (2) vertex and most of genae marked with numerous yellowish or pale brown speckles; (3) cephalic process relatively broad and long; (4) vertex with median longitudinal carina obsolete, posterior region obviously higher than pronotum; (5) legs moderately long, fore femur with a short and blunt spine near apex; hind tibiae with 5 or 6 lateral black-tipped spines and 8 apical black-tipped teeth, spinal formula 8/(9–12)/(9–12); (6) aedeagus with a pair of phallobasal conjunctival processes apically and phallobase sclerotized and pigmented, with two membranous lobes apically.

###### Distribution.

China (Fujian, Guangdong, Guangxi, Heilongjiang, Henan, Hubei, Hunan, Jilin, Jiangxi, Shaanxi, Sichuan, Taiwan, Yunnan, Zhejiang, Gansu, Guizhou); Indo-China; Japan (Hokkaido, Honshu); Russia (Primorye, Far East); Korea (South).

### Key to species of the genus *Saigona*

(Modified from Liang and Song (2006), as updated by Zheng et al. 2014).

**Table:** 

1	Vertex with cephalic process short, shorter than pronotum and mesonotum combined	**2**
–	Vertex with cephalic process long, longer than or nearly as long as pronotum and mesonotum combined	**7**
2	Postclypeus yellowish or yellowish brown	**3**
–	Postclypeus fuscous	**5**
3	Mesonotum with a yellowish stripe along median longitudinal carina	**4**
–	Mesonotum without a yellowish stripe along median longitudinal carina (Zheng et al. 2014: fig. 51)	***S.dicondylica* Zheng, Yang & Chen**
4	Pygofer short and broad in lateral aspect, posterior margin straight and angularly excavated at apical 1/4 apex to accommodate anal tube, aedeagus with phallobase having apical ventral membranous lobe with numerous fine spines at apex (Liang & Song, 2006: fig. 80, fig. 84)	***S.ussuriensis* (Lethierry)**
–	Pygofer large and broad in lateral aspect, posterior margin nearly straight, and gently excavated at apical 1/3 to accommodate anal tube, aedeagus with phallobase having apical dorsal and ventral membranous lobes with numerous fine spines at apex (Liang and Song 2006: figs 50, 54)	***S.latifasciata* Liang & Song**
5	Frons with lateral carinae not reaching frontoclypeal suture (Liang and Song 2006: fig. 58)	***S.fuscoclypeata* Liang & Song**
–	Frons with lateral carinae almost reaching frontoclypeal suture	**6**
6	Aedeagus with phallobasal conjunctival processes spiraled dorsally (Liang and Song 2006: fig. 44)	***S.henanensis* Liang & Song**
–	Aedeagus with phallobasal conjunctival processes not spiraled dorsally (Zheng et al. 2014: fig. 10)	***S.anisomorpha* Zheng, Yang & Chen**
7	Cephalic process bulbous apically, with 3 pairs of symmetrical knob-like protuberance on lateral regions	**8**
–	Cephalic process not bulbous apically, without knob-like protuberance on lateral regions	**10**
8	Aedeagus with phallobasal conjunctival processes not produced out of phallobase (Zheng and Chen 2011: fig. 10)	***S.saccus* Zheng & Chen**
–	Aedeagus with phallobasal conjunctival processes produced out of phallobase	**9**
9	Pygofer posterior margin with an elongate, acute process on dorsocaudal margin (Liang and Song 2006: fig. 20); phallobase with membranous lobe simple round in ventral view (Liang and Song 2006: fig. 23)	***S.fulgoroides* (Walker)**
–	Pygofer posterior margin without an alongate process on dorsocaudal margin (Fig. 8); phallobase with membranous lobe complex in ventral view (Fig. 13)	***S.baiseensis* Zheng & Chen, sp. nov.**
10	Tip of cephalic process with a yellow spot	**11**
–	Tip of cephalic process without a yellow spot	**12**
11	Frons with lateral carinae almost reaching frontoclypeal suture (Liang and Song 2006: fig. 15)	***S.capitata* (Distant)**
–	Frons with lateral carinae reaching the eyes, but not frontoclypeal suture, pygofer posterior margin with a slightly blunt process dorsally (Fig. 25); aedeagus with phallobasal conjunctival processes unequal in length (Fig. 28)	***S.maculata* Zheng & Chen, sp. nov.**
12	Mesonotum with very narrow, yellowish stripe along median longitudinal carina	***S.taiwanella* Matsumura**
–	Mesonotum with broad, yellowish stripe along median longitudinal carina	**13**
13	Posterior margin of pygofer produced into a large process dorsally (Zheng et al. 2014: fig. 40)	***S.tenuisa* Zheng, Yang & Chen**
–	Posterior margin of pygofer not produced into a large process dorsally	**14**
14	Aedeagus with phallobase having apical dorsal and ventral membranous lobes (Liang and Song 2006: fig. 74)	***S.sinicola* Liang & Song**
–	Aedeagus with phallobase having apical ventral membranous lobes	**15**
15	Aedeagus with phallobasal conjunctival processes subparallel apically (Zheng et al. 2014: fig. 21)	***S.daozhenensis* Zheng, Yang & Chen**
–	Aedeagus with phallobasal conjunctival processes diverging apically (Liang and Song 2006: fig. 63)	***S.robusta* Liang & Song**

#### Saigona baiseensis

Taxon classificationAnimaliaHemipteraDictyopharidae

Zheng & Chen sp. nov.

00004033-2F28-53C2-9573-4E7BD4190F05

http://zoobank.org/BC9FDBB3-4918-4E0C-B875-D242F91DE8AA

Figures 1 , 2–5 , 6–13 , 14–18 , 19–20

##### Type locality.

Tianlangping Baise, Guangxi Zhuang Autonomous Region, China.

##### Diagnosis.

This species can be distinguished from other *Saigona* species by the combination of the following diagnostic characters: (1) pygofer large and broad in lateral view, posterior margin with a blunt dorsal process; (2) aedeagus with phallobasal conjunctival processes unequal in length; (3) phallobase narrow and long, curved dorsally, with 2 apical membranous dorsal apical lobes (Fig. 12), dorsal round and large; ventral lobes (Fig. 13) small and slender, with another small membranous lobe on it.

##### Description.

***Measurement*.** ♂, BL: 17.4–18.9 mm; HL: 1.5–1.6 mm; HW: 1.4–1.5 mm; FWL: 11.3–13.2 mm. ♀, BL: 18.9–20.2 mm; HL: 1.6–1.7 mm; HW: 1.4–1.5 mm; FWL: 13.2–14.6 mm.

**Figures 2–5.**
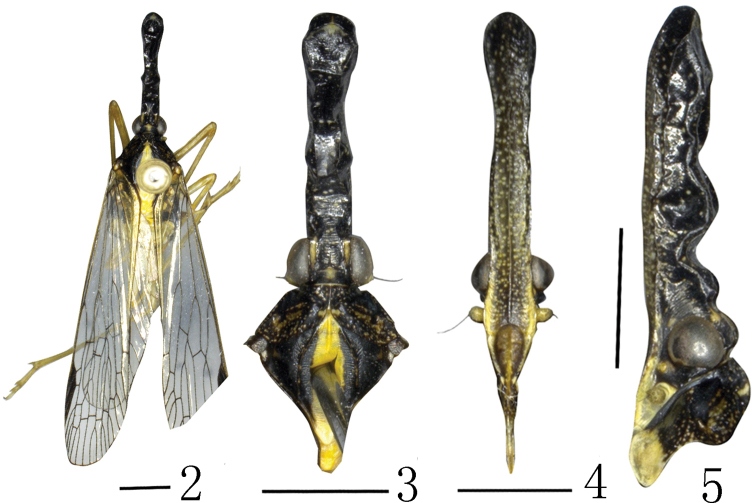
*Saigonabaiseensis* Zheng & Chen sp. nov. **2** male, holotype, dorsal view **3** male, head and thorax, dorsal view **4** male, head, frons and clypeus, lateral view **5** male, head and pronotum, lateral view. Scale bars: 2 mm (**2–5**).

***Coloration*.** General color dark, marked with fuscous and ochraceous (Figs 2–5). Vertex brown with median carina very faint, lateral margins dark. Genae dark, yellow ventroposteriorly near antennae (Fig. 4). Eyes dark brown, lateral ocelli yellowish, antenna brown and areas surrounding ocellus and antenna beneath eye yellowish. Frons dark brown with yellowish speckles (Fig. 3). Postclypeus and anteclypeus pale brown. Pronotum dark with scattered white speckles; mesonotum dark, with broad median longitudinal yellowish stripe. Ventral thorax and fore femur dark, other areas yellow. Legs ochraceous except coxae which are dark. Forewing venation brown and pterostigma dark. Abdomen dark with scattered white speckles and median longitudinal yellowish stripe. Male genitalia black.

**Figures 6–13.**
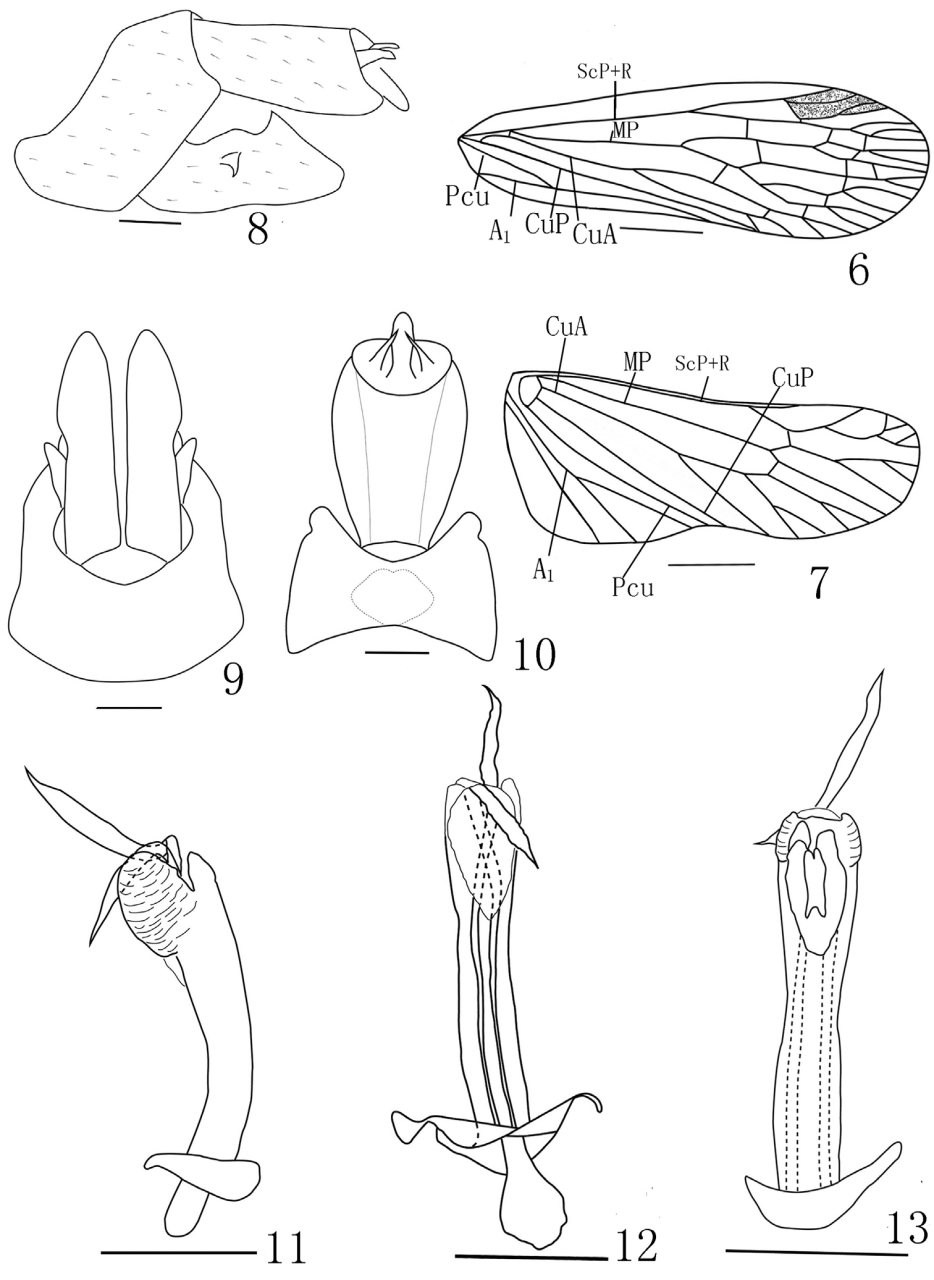
*Saigonabaiseensis* Zheng & Chen, sp. nov. **6** forewing **7** hindwing **8** genitalia, lateral view **9** pygofer and gonostyli, ventral view **10** pygofer and anal tube, dorsal view **11** aedeagus, lateral view **12** aedeagus, ventral view **13** aedeagus, dorsal view. Scale bars: 2 mm (**6–10**), 0.5 mm (**11–13**).

***Head and thorax*.** Head (Figs 2–5) longer than pronotum and mesonotum combined (1.45:1). Vertex (Fig. 3) with median carina very faint, only conspicuous at apex and base; lateral carinate margins sinuate. Frons (Fig. 3) with lateral carinae reaching to front of eyes. Pronotum (Figs 2, 3, 5) with median carina distinct, lateral carinae very faint. Mesonotum (Figs 1, 2) tricarinate on disc, lateral carinae curved towards median carinate at the front. Fore wings (Figs 2, 6) hyaline with ScP+R, MP and Cu branched apically, longer than widest part, with length to maximum width ratio of 3.0; stigma distinct, with 3 or 4 cells, veins with numerous fuscous setae. costal margin distinctly expanded into a narrow, sclerotized costal area, without transverse veins; ScP+R branched apically; MP bifurcating into MP_1+2_ and MP_3+4_ at basal 3/5 and beyond CuA; CuA forked near middle of forewing; 13 apical marginal cells between RP and CuA; Pcu and A_1_ fusing at apical 2/5 of clavus. Hindwings (Fig. 7) well developed, legs moderately elongate, fore femora flattened and dilated; hind tibiae with 5 or 6 lateral black-tipped spines and 8 apical black-tipped teeth, spinal formula 8/(10–12)/(10–12).

**Male *genitalia*.** Pygofer (Figs 8–10) large and broad in lateral view, posterior margin with a rounded lobe at level of venter of anal tube. Gonostyli (Figs 8, 9) relatively large and broad, apex sharply rounded, protruded posteriorly in lateral view on the outer surface of the gonostyli (Fig. 8). Aedeagus (Fig. 11) with phallobasal conjunctival processes unequal in length, left one obviously longer than right one; phallobase narrow and long, curved dorsally, with 2 apical membranous dorsal apical lobes (Fig. 12) dorsal round and large; ventral lobes (Fig. 13) small and slender, with another small membranous lobe on it. Segment X large in lateral view (Fig. 8), large, long, ovoid in dorsal view (Fig. 10), ratio of length to width at middle about 1.5.

**Figures 14–18.**
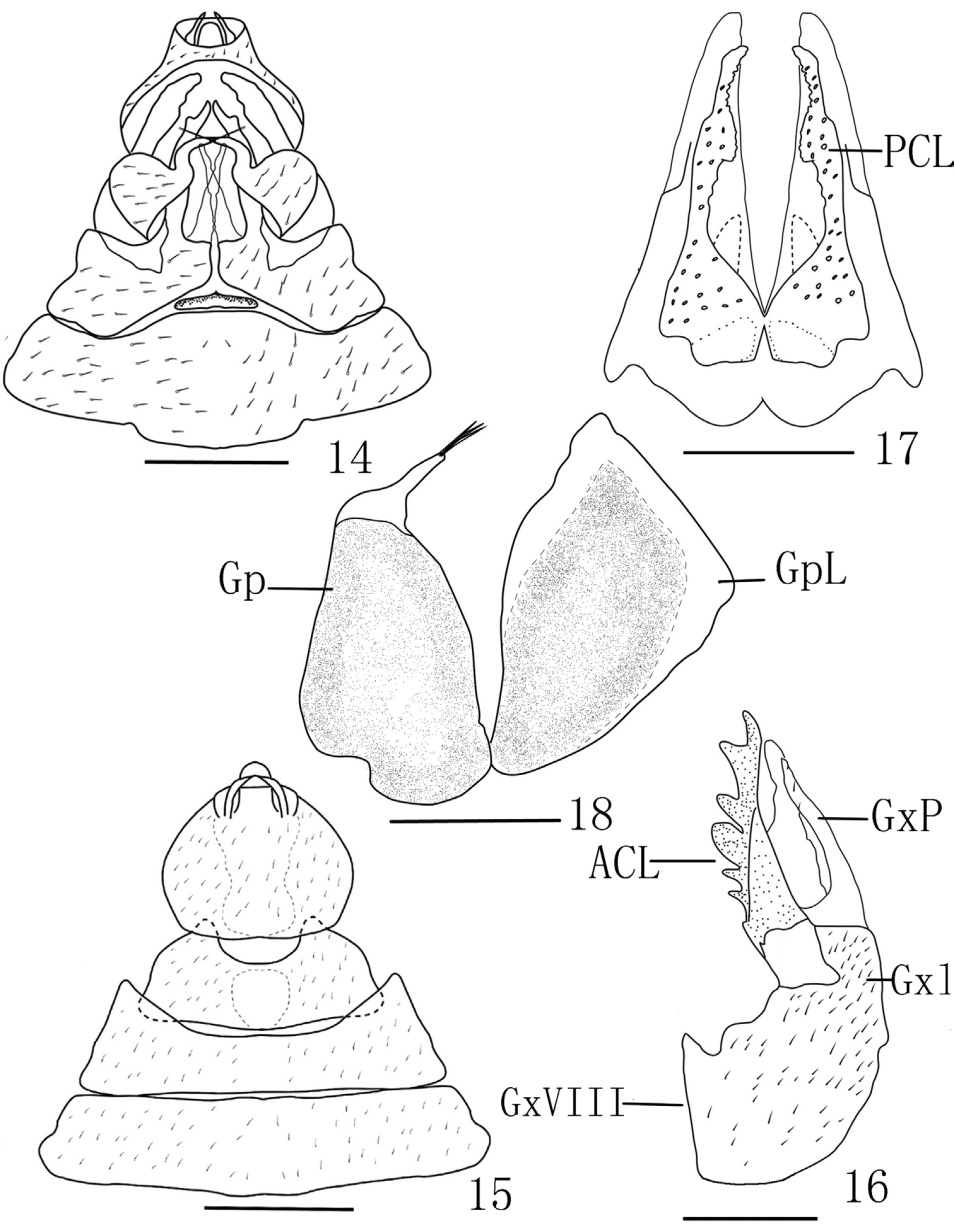
*Saigonabaiseensis* Zheng & Chen, sp. nov. **14** genitalia of female, ventral view **15** genitalia of female, dorsal view **16** anterior connective lamina of gonapophysis VIII (lateral view) **17** gonapophysis IX (ventral view) **18** gonoplacs (lateral view). Scale bars: 1 mm (**14, 15**), 0.5 mm (**16–18**). Gp, gonoplacs; Gx VIII, gonocoxae VIII; GxL, gonocoxae VIII with endogonocoxal lobe; GxP, gonocoxae VIII with endogonocoxal process; PCL, posterior connective lamina of gonapophysis IX; GpL, posterior lobe of the gonoplac; ACL, anterior connective lamina of gonapophyses.

**Female *genitalia*** (Figs 14, 16) with gonocoxae VIII with GxP membranous and flattened (Fig. 16). Gonopophyses VIII with anterior connective lamina of gonapophyses (ACL) moderately sclerotized with 7 unevenly sized teeth in lateral view. (Fig. 16). Gonopophyses IX (Fig. 17) with posterior connective lamina of gonapophysis IX (PCL) triangular, symmetrical in ventral view, connected at base and separated from 1/3 base. Gonoplacs (Fig. 18) with 2 sclerotized lobes: gonoplacs (Gp), with 3 or 4 long spines at apex, and posterior lobe of the gonoplac (GpL) with membranous structure at top. Segment X in dorsal view relatively round and large, with ratio of length to width at middle about 0.8 (Fig. 15).

##### Type material.

***Holotype*** ♂, Tianlangping Baise, Guangxi Zhuang Autonomous Region, 24 Apr. 2012, Weicheng Yang. ***Paratypes***, 8♂♂2♀♀, same to holotype, Weibin Zheng, Zaihua Yang, Jiankun Long.

##### Etymology.

This new species is named for the type locality, Baise City, Guangxi, China.

##### Distribution.

China (Guangxi).

##### Remarks.

This species is similar to *S.fulgoroides* (Walker, 1858) (Liang and Song 2006: figs 20, 23–25) but can be distinguished by the large, broad pygofer (in lateral view) with its posterior margin bearing a somewhat blunt process dorsally (vs sharp, dorsoposteriorly directed process near apex in *S.fulgoroides*); the aedeagus has a conjun

#### Saigona maculata

Taxon classificationAnimaliaHemipteraDictyopharidae

Zheng & Chen sp. nov.

398D4D01-6DD5-529B-8802-FFC173CCEF33

http://zoobank.org/74E6E965-788E-4065-BE18-DB7C5C2A9871

Figures 19–22 , 23–31

##### Type locality.

Lang Ping town, Tianlin County, Guangxi Zhuang Autonomous Region, China.

##### Diagnosis.

(1) Head moderately long, longer than pronotum and mesonotum combined. Cephalic process relatively long and robust, somewhat upturned; (2) pygofer with posterior margin sinuate in lateral view; (3) aedeagus with phallobasal conjunctival processes unequal in length.

##### Description.

***Measurement*.** ♂, BL: 15.7 mm; HL: 2.1 mm; HW: 0.8 mm; FWL: 11.8 mm.

**Figures 19–22.**
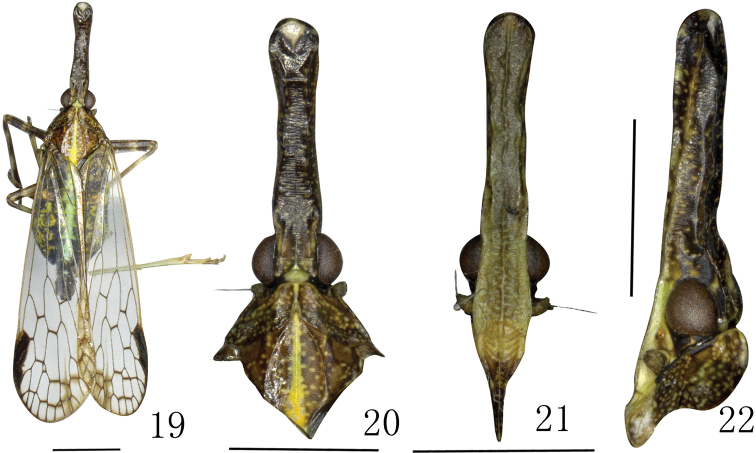
*Saigonamaculata* Zheng & Chen sp. nov. **19** male, holotype, dorsal view **20** male, head and thorax, dorsal view **21** male, frons and clypeus, ventral view **22** male, head and pronotum, lateral view. Scale bars: 2 mm (**19–22**).

***Coloration*.** General color dark brown, marked with fuscous and ochraceous speckles (Figs 19–22). Vertex dark brown with a yellowish green spot at top. Genae brown, eyes brown, ocellus yellowish, antenna brown and areas surrounding ocellus and antenna beneath eye yellowish. Frons yellowish brown. Postclypeus and anteclypeus yellow. Pronotum brown with median carina yellowish; lateral, ventrally curved areas yellowish. Mesonotum fuscous, with a narrow, yellow stripe along median longitudinal carina. Abdomen fuscous, scattered white speckle, with median longitudinal green stripe. Forewing venation brown and stigma dark. Legs ochraceous. Genitalia black.

***Head and thorax*.** Head (Figs 19–22) moderately long, longer than pronotum and mesonotum combined (5.1:1). Cephalic process relatively long and robust, somewhat upturned. Vertex (Fig. 20) with median carina very faint, only conspicuous at base, lateral carinate margins curved in front of eyes. Frons (Fig. 21) with lateral carinate reaching to the front of eyes, not to frontoclypeal suture. Pronotum (Figs 19, 20, 22) with median carina distinct, lateral carinae very faint; mesonotum tricarinate on disc, lateral carinae curved towards median carinae at front.

**Figures 23–31.**
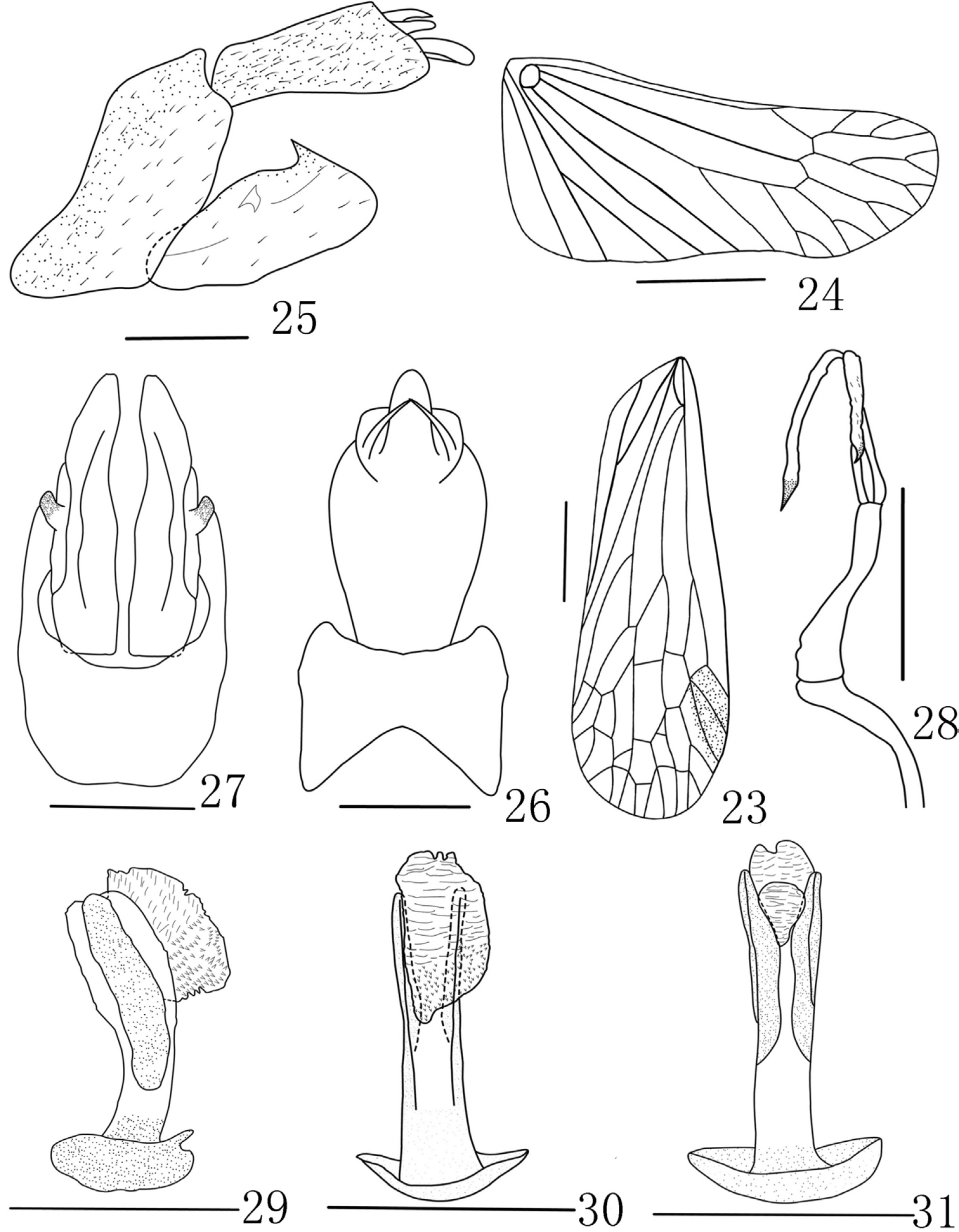
*Saigonamaculata* Zheng & Chen sp. nov. **23** forewing **24** hindwing **25** genitalia, lateral view **26** pygofer and anal tube, dorsal view **27** pygofer and gonostyles, ventral view **28** aedeagus process **29** aedeagus, lateral view **30** aedeagus, ventral view **31** aedeagus, dorsal view. Scale bars: 2 mm (**22–30**), 0.5 mm (**28–34**).

***Fore wings*** (Figs 19, 23) hyaline with Sc+R, M and Cu branched apically, longer than widest part, with length to maximum width ratio of 3.0; pterostigma distinct, with 2 cells, veins with numerous fuscous setae. costal margin distinctly expanded into a narrow, sclerotized costal area, without transverse veins; ScP+R branched apically; MP bifurcating MP_1+2_ and MP_3+4_ at basal 3/5 and beyond CuA; CuA forked into two branches near middle of forewing 14 apical cells between RP and CuA; Pcu and A_1_ fusing in apical 2/5 of clavus. Hindwings (Fig. 24) well developed. Legs moderately elongate, fore femora flattened and dilated; hind tibiae with 5 lateral black-tipped spines and 8 apical black-tipped teeth, spinal formula 8/(9–11)/(10–12).

**Male *genitalia*.** Pygofer (Figs 25–27) in lateral view with posterior margin slightly sinuate. Gonostyli (Figs 25, 27) relatively large, broad in lateral view (Fig. 25), apex sharply rounded, apex sharply rounded, protruded posteriorly in lateral view on the outer surface of the gonostyli. Aedeagus with phallobasal conjunctival processes unequal in length, left one obviously longer than right one (Fig. 28); phallobase narrow and long, curved dorsally, with 2 apical membranous dorsal apical lobes (Fig. 31) sclerotized on both sides with circular membranous processes in the middle; ventral lobes (Fig. 30) large, membranous fold, with spines at base. Segment X (Figs 25, 26) large, nearly triangular in lateral view; round in dorsal view, ratio of length to width at middle about 1.5:1.

**Female.** unknown.

##### Type material.

***Holotype*** ♂, Lang Ping town, Tianlin County, Guangxi Zhuang Autonomous Region, 23 Apr. 2012, Zaihua Yang. ***Paratypes***, 1♂, same as holotype.

##### Etymology.

The name of the new species is derived from the Greek word *maculata* (spotted), in reference to the vertex with a yellowish green spot at the apex.

##### Distribution.

China (Guangxi).

##### Remarks.

This species is similar to *S.tenuisa* Zheng, Yang & Chen, 2014 but can be distinguished from the latter by the pygofer with the posterior margin slightly sinuate in lateral view and the aedeagus with the phallobasal conjunctival processes unequal in length.

## Discussion and conclusions

Species of *Saigona* are externally similar to those of *Leprota* Melichar, 1912, but *Leprota* can be separated from *Saigona* by the following: 1) body generally rust-brown or rust-red above, without pale speckles in *Leprota* (vs ochraceous or fuscous, with pale speckles on the vertex and most of the genae in *Saigona*); 2) head covered in numerous irregular transverse wrinkles in *Leprota* (vs not covered irregular transverse wrinkles, head long and broad, distinctly produced into a cephalic process, vertex with lateral margins carinate, sinuate in front of eyes in *Saigona*); 3) forewings elongate, with numerous netted veins on apical 1/5 in *Leprota* (vs relatively short, with sparse netted veins on apical area in *Saigona*); and 4) the fore femora normal in *Leprota* (the fore femora flattened and dilated, with short and blunt spine near apex in *Saigona*) (Song et al. 2012).

**Figure 32.**
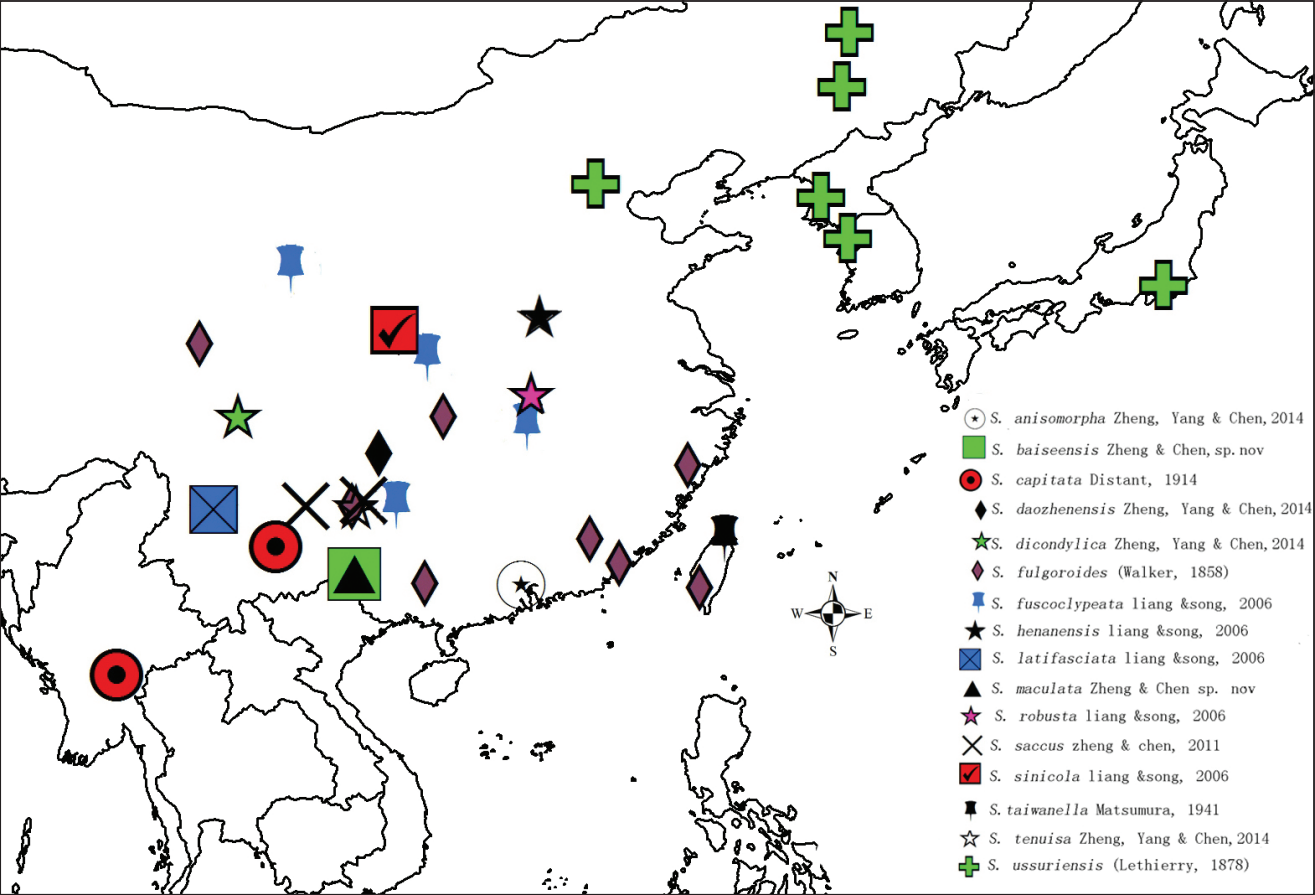
Geographical distribution of the *genus Saigona* species.

The distribution of the genus is quite restricted (Fig. 32), extending from the north-eastern Sino-Japanese to north-eastern Oriental realms. *Saigonaussuriensis* is widely distributed in the north but not crossing into the Palearctic realm, and *S.henannensis*, *S.fuscoclypeata*, *S.sinicola* and *S.robusta* are Sino-Japanese. All other species occur in south and eastern continental China and can be considered as Oriental; *S.fulgoroides* and *S.taiwanella* from Taiwan are also in the Oriental group of species. One species, *S.capitata* from South Korea provides the south and western limits of the genus. Absent from the Palearctic and India, and wrongly reported from Indonesia (Sumatra, Borneo) (Song et al. 2012), *Saigona* is almost exclusively a Chinese endemic genus. However, the species diversity observed in this genus suggests that the discovery of additional species in the Indochinese peninsula cannot be excluded.

## Supplementary Material

XML Treatment for Saigona

XML Treatment for Saigona baiseensis

XML Treatment for Saigona maculata
